# Molecular evolution of bovine Toll-like receptor 2 suggests substitutions of functional relevance

**DOI:** 10.1186/1471-2148-8-288

**Published:** 2008-10-20

**Authors:** Oliver C Jann, Dirk Werling, Jung-Su Chang, David Haig, Elizabeth J Glass

**Affiliations:** 1 The Roslin Institute and Royal (Dick) School of Veterinary Studies, University of Edinburgh, Roslin Biocentre, Midlothian, EH25 9PS, UK; 2 Department of Pathology and Infectious Diseases, The Royal Veterinary College, Hawkshead Lane, Hatfield, Herts AL9 7TA, UK; 3 Division of Virology, Moredun Research Institute, Pentland Science Park, Edinburgh, EH26 OPZ, UK; 4 School of Veterinary Medicine and Science, Nottingham University, Sutton Bonington LE12 5RD, UK

## Abstract

**Background:**

There is accumulating evidence that polymorphism in Toll-like receptor (*TLR) *genes might be associated with disease resistance or susceptibility traits in livestock. Polymorphic sites affecting TLR function should exhibit signatures of positive selection, identified as a high ratio of non-synonymous to synonymous nucleotide substitutions (ω). Phylogeny based models of codon substitution based on estimates of ω for each amino acid position can therefore offer a valuable tool to predict sites of functional relevance. We have used this approach to identify such polymorphic sites within the bovine *TLR2 *genes from ten *Bos indicus *and *Bos taurus *cattle breeds. By analysing *TLR2 *gene phylogeny in a set of mammalian species and a subset of ruminant species we have estimated the selective pressure on individual sites and domains and identified polymorphisms at sites of putative functional importance.

**Results:**

The ω were highest in the mammalian TLR2 domains thought to be responsible for ligand binding and lowest in regions responsible for heterodimerisation with other TLR-related molecules. Several positively-selected sites were detected in or around ligand-binding domains. However a comparison of the ruminant subset of *TLR2 *sequences with the whole mammalian set of sequences revealed that there has been less selective pressure among ruminants than in mammals as a whole. This suggests that there have been functional changes during ruminant evolution. Twenty newly-discovered non-synonymous polymorphic sites were identified in cattle. Three of them were localised at positions shaped by positive selection in the ruminant dataset (Leu227Phe, His305Pro, His326Gln) and in domains involved in the recognition of ligands. His326Gln is of particular interest as it consists of an exchange of differentially-charged amino acids at a position which has previously been shown to be crucial for ligand binding in human TLR2.

**Conclusion:**

Within bovine TLR2, polymorphisms at amino acid positions 227, 305 and 326 map to functionally important sites of TLR2 and should be considered as candidate SNPs for immune related traits in cattle. A final proof of their functional relevance requires further studies to determine their functional effect on the immune response after stimulation with relevant ligands and/or their association with immune related traits in animals.

## Background

The innate immune system is the first line of host defence against pathogens and is activated by host recognition of conserved pathogen-associated molecular patterns (PAMPs). These engage families of pattern-recognition receptors (PRRs) that include the Toll-like receptors (TLRs) [1]. The TLRs form an ancient gene group which is found in invertebrates and vertebrates and there are related genes in plants [2]. In vertebrates, six TLR families of common ancestry and similar recognition patterns have been identified. The TLR1 family consists of TLR1, 2, 6 and 10 and is involved in the recognition of gram-positive and gram-negative bacteria [3]. TLR1 and 6 form heterodimers with TLR2, a crucial process for identification of several PAMPs [4,5]. TLR2 recognises both tri- and di-acylated lipopeptides whereas TLR1 and TLR6 recognise tri- and di-acylated lipopeptides, respectively [4,6,7]. In mammals at least ten of these *TLR *genes have been described, each binding different PAMPs representing a wide range of pathogens.

The TLRs consist of a large extracellular domain responsible for PAMP binding, a transmembrane domain and an intracellular Toll/interleukin-1 receptor (TIR) domain which binds signalling molecules and initiates innate cellular immune responses [8].

The extracellular domains are composed of approximately 20 leucine-rich repeats (LRRs), motifs of 20–30 amino acids (AA) in length, forming a solenoid shape and potentially binding the TLR specific patterns. TLR2 binds its ligands either on its concave or convex surfaces [9].

The concave surface is formed by a highly conserved segment (HCS) consisting of 11 or 12 AA, normally following a pattern LxxLxLxxNxL or LxxLxLxxCxxL. The convex side is shaped by α-helical elements from the AAs of the variable segment (VS) [10,11]. The TIR domain interacts with adapter proteins that direct the intracellular signalling cascade after TLR-PAMP engagement. Substitutions within the TIR domain, particularly in the BB loop have been shown to impact on receptor signalling [12], while mutagenesis of the DD loop identified residues essential for TLR1/2 dimerisation [13].

Due to their central role in host defence, polymorphisms within TLR genes are associated with predisposition to a range of diseases in humans and mice. Accumulating evidence suggests that polymorphism in *TLR *genes is also associated with variations in disease resistance traits in livestock [14,15]. An Arg753Gln polymorphism in TLR2 has been associated with a predisposition to staphylococcal infection [16], tuberculosis [17], rheumatic fever [18] and urinary tract infection in children [19]. TLRs are under strong selection for both maintenance and adaptation of function [3]. The intracellular TIR domain is highly conserved with functional similarity among species and TLR genes, as it is involved in engaging signalling pathways within cells [20]. However, the extracellular TLR domains exhibit significantly-higher divergence reflecting their involvement in PAMP recognition from multiple microbial sources. Amino acid changes are concentrated in the region between LRR10 and LRR15, that is important for PAMP ligand binding [21]. TLR1 and TLR6 phylogeny is also driven by co-evolution caused by gene conversion which could also affect their heterodimerisation [22].

Protein evolution is shaped by divergent selection pressures on sites of different functional relevance. This process can be reproduced by phylogeny-based models of codon substitution which are based on a site-by-site analysis of the non-synonymous (d_N_)/synonymous (d_S_) substitution ratio (ω) between sequences [23,24]. In many cases protein function is maintained against occurring disadvantageous mutations by purifying selection resulting in high conservation of such sites or domains (ω < 1). In contrast, the adaptive evolution of protein function can be driven by positive selection of advantageous mutations at functionally-important sites (ω > 1). This has been observed in genes involved in immune responses to pathogens, not just particularly the MHC complex [25], but also in Toll-like receptor genes [26].

Such phylogeny-based models can offer a valuable tool to predict sites of putative functional relevance [27-29]. However, it is also possible that sites with high ω might reflect ancient adaptive selection and the functional relevance of certain sites could have changed with the adaptation process [30].

In this study, we take advantage of a phylogeny based approach to identify bovine *TLR2 *polymorphisms revealed by analysing the gene in ten predominant *Bos indicus *and *Bos taurus *cattle breeds. Through the analysis of the *TLR2 *phylogeny in a set of mammalian species we will estimate the selective pressure on individual bovine TLR2 sites and domains. To detect variations in selective constraint during evolution caused by possible functional changes we will compare our results with a subset of *TLR2 *sequences from ruminants only.

## Results

### TLR2 sequences and domain structure

The length of the predicted TLR2 proteins in all species analysed ranged between 784 – 786 AAs. In all species, TLR2 shared a common domain architecture: an extra-cellular domain containing 20 Leucine Rich Repeats between AA54 and AA584, a transmembrane domain at AA585–AA607 and an intracellular TIR domain at AA633–AA783.

### Selective constraint and sequence conservation

In some domains of TLR2 sequence variation (corrected entropy, see additional file 1: Comparative plot of the average corrected entropy of a 50 AA sliding window along the protein sequence) was high, particularly in the region between AA200 and AA310, comprising LRR 7–10. Most of the other domains were more conserved, particularly LRR 12 and LRR 13 (AA340–AA390) and the TIR domain (AA610-end).

To interpret these results with respect to domain and AA-specific selective constraint, the sequences were subjected to Phylogenetic Analysis by Maximum Likelihood (PAML). The average ω over the whole TLR2 protein using data from all the mammalian sequences was 0.45. The model assigned all AA sites into three classes of 461, 206 and 120 sites with an average ω of: 0.05 (class1: low ω, purifying selection), 0.61 (class 2: intermediate ω, nearly neutral), 1.70 (class 3: high ω, potentially positive selection) and average probabilities for the class allocation of 0.96, 0.90 and 0.88, respectively. Amino acid sites in the latter class are likely to be under positive selection if their ω is > 1 with high probability. This is true for 69 out of the 120 AA sites in this class with at least 95% probability (Table 1).

**Table 1:** Significantly positive selected sites

Position	AA	P	ω	Position	AA	P	ω
**3**	R	0.98591	1.84	**279**	I	0.99157	1.847
**7**	T	0.97386	1.824	**296**	R	0.99915	1.857
**8**	A	0.98487	1.838	**297**	A	0.99833	1.856
**20**	G	0.9561	1.801	**298**	L	0.99039	1.846
**21**	A	0.99537	1.852	**299**	S	1	1.858
**23**	D	0.99588	1.853	**300**	L	1	1.858
**32**	P	0.9598	1.806	**302**	R	0.99562	1.852
**33**	T	0.96925	1.818	**304**	R	0.99646	1.854
**39**	H	0.99755	1.855	**305**	H	0.99983	1.858
**52**	A	0.99338	1.85	**306**	L	0.99544	1.852
**74**	R	0.96928	1.818	**326**	H	0.9823	1.835
**82**	R	0.99748	1.855	**336**	G	0.96614	1.814
**84**	G	0.99999	1.858	**337**	R	0.97197	1.821
**98**	H	0.98764	1.842	**373**	S	0.99991	1.858
**111**	R	0.99686	1.854	**376**	T	0.99962	1.858
**125**	V	0.99792	1.855	**384**	D	0.99406	1.85
**135**	L	0.99946	1.857	**400**	K	0.99999	1.858
**149**	P	0.98322	1.836	**408**	L	0.99983	1.858
**159**	S	0.99998	1.858	**440**	Q	0.99993	1.858
**166**	H	0.98063	1.833	**453**	Q	1	1.858
**167**	E	0.99467	1.851	**471**	S	0.99083	1.846
**182**	S	0.99939	1.857	**502**	S	0.99786	1.855
**184**	Q	0.99998	1.858	**508**	R	0.98935	1.844
**188**	I	0.99979	1.858	**510**	I	0.99123	1.847
**221**	V	1	1.858	**512**	N	0.96372	1.811
**235**	H	0.99283	1.849	**523**	Q	0.9997	1.858
**242**	A	0.99239	1.848	**524**	Q	0.99985	1.858
**244**	I	0.99197	1.848	**553**	R	0.99999	1.858
**245**	S	0.99752	1.855	**556**	V	0.98053	1.833
**247**	M	0.99991	1.858	**560**	D	0.99864	1.856
**250**	S	0.98942	1.844	**561**	D	0.99969	1.858
**260**	Q	0.99472	1.851	**636**	R	0.99989	1.858
**267**	V	0.99995	1.858	**650**	R	0.96011	1.806
**274**	N	0.95099	1.794	**766**	V	0.9999	1.858
**275**	Y	0.98963	1.845				

Position: position in the protein sequence, AA: amino acid at this position in cattle, p: probability of class assignment as positively selected site, ω: dN/dS ratio

**Figure 1:**
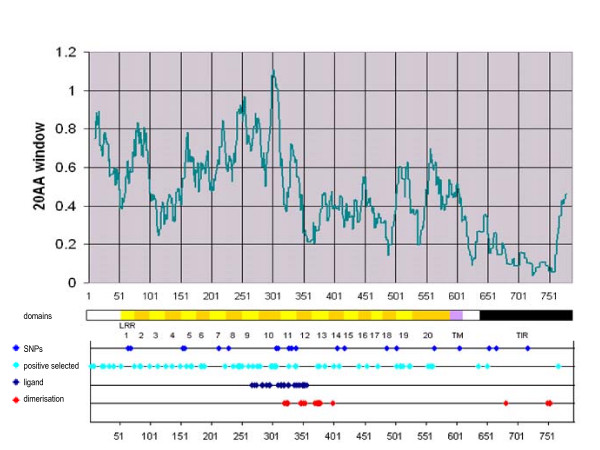
**Average dN/dS ratio of a 20 AA sliding window along the protein sequence**. The analysis is based on the complete dataset of 22 mammalian sequences. The predicted domain structure, position of polymorphic and positively selected (*P *> 0.95) sites is indicated. Sites displayed as "functional" refer to positions which were found to be crucial for ligand binding or heterodimerisation in human (additional file 7: Functional relevant TLR2 sites identified in former studies in human or mouse).

The ω in the extracellular domain were mostly higher than those in the intracellular domains (Figure 1). The most divergent regions were between AA11–AA17 (signal peptide), AA241–AA255 (VS of LRR8), AA268–AA277 (VS of LRR9) and AA294–AA313 (VS of LRR10) with average ω of the surrounding 20 AA of > 0.8. In the region between AA296–AA306 (LRR10) nine of the 10 AA are significantly under positive selection (*P *> 0.95) with ω over 1.8 for all of them.

The highest conserved regions of the extracellular domain were at AA348–AA374 (VS of LRR12 and HCS of LRR13), AA482–AA494 (HCS of LRR18) and AA534–AA543 (HCS of LRR20) with average ω of the surrounding 20 AA smaller than 0.3. In these regions there were also several interspersed sites that were likely to evolve under positive selection with *P *> 0.95 (Table 1).

The TLR2 intracellular domains were generally much more conserved through strong purifying selection, reaching maximum conservation in the TIR domain with ω < 0.1 for the vast majority of sites. Nevertheless, interspersed within this highly conserved domain several AA sites were assigned to class 3 with high probabilities (*P *> 0.95) suggesting that they are under positive selection (Positions 639, 650, 760). A list of class assignment and probabilities for all AA sites is provided in the additional file 2: Class assignment of AA sites.

### Taxon-specific selected sites

Ruminant-specific ω (average 0.446) were very similar to those estimated in the dataset that included all mammalian species analysed (0.450). Nevertheless, selective constraint appears to have acted differently on some AAs and domains. Forty-four AA sites in ruminants were assigned to class 3 (high ω), 740 to class 2 and none to class 1, with only 10 positively-selected AA sites as significant (*P *> 0.95). From the 44 class 3 AA sites in ruminants, 27 were assigned to class 3 in all mammals. Only 215 sites were assigned to the same class of selective constraint in ruminants as well as in all mammals. 475 AA sites differed by one class (e.g. negatively selected *versus *neutral or positively selected *versus *neutral) and one site differed by two classes (positive selection *versus *purifying selection) in ruminants *versus *all mammals (227).

Selective pressure has acted differentially on several domains of the ruminant TLR2 than in mammals in general (additional file 3: Comparative plot of the average ω of a 20 AA sliding window along the protein sequence). Eight LRR domains, LRR2 (ω difference (diff): -0.42), LRR5 (diff: -0.18), LRR7 (diff: -0.17), LRR8 (diff: -0.32), LRR9 (diff: -0.43), LRR10 (diff: -0.17), LRR11 (diff: -0.14) and LRR19 (diff: -0.16), were more conserved within ruminants than within all mammalian species studied. This contrasted with LRR12 (diff: 0.23), 13 (diff: 0.14) and TIR (diff: 0.23), which were more diverse within ruminants when compared to the complete mammalian species dataset (additional file 2: Class assignment of AA sites).

### Phylogenetic trees of TLR2 in mammals

All constructed trees reflect, in general, the putative evolution of the analysed species. However, two exceptions to this generalisation were observed: a closer relationship between *Capra ibex *and *Ovis aries *than between *Capra ibex *and *Capra hircus *and a closer relationship between *Boselaphus tragocamelus *and *Bos taurus *than between *Bubalus bubalis *and *Bos taurus*.

Trees based on synonymous exchanges clustered equally to those based on non-synonymous exchanges (additional file 4: Unrooted radial phylogenetic trees of the analysed TLR2 sequences). Nevertheless, they differed in relative branch lengths indicating branch specific rates of evolutionary change of the TLR2 protein sequence. This was reflected in different ω along branches (additional file 5: ω along tree branches). Specifically, along most (28 out of 42) branches the TLR2 sequence evolved under similar selective pressure and with ω between 0.2–0.6. In four branches however, low ω (0–0.16) indicated low evolutionary change (conserved branches), while in 10 branches high ω (0.86-infinity) were generated by relatively very high numbers of non-synonymous exchanges.

### Branches evolving under positive selection

Seven of the 10 branches with high ω were short with less than 0.01 exchanges per codon (additional file 4: Unrooted radial phylogenetic trees of the analysed TLR2 sequences). Their evolutionary rate is consequently difficult to assess and could have been caused by chance. These branches are therefore not further considered here. Three further branches with high ω were much longer and suggested an accelerated evolutionary rate caused by positive selection on certain domains.

**Figure 2:**
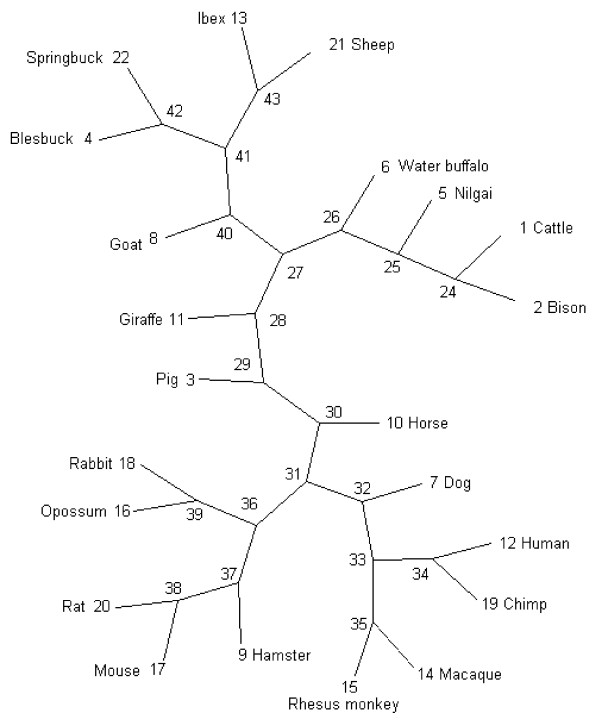
**Schematic phylogenetic tree of the TLR2sequences**. Numbers represent nodes and are discussed in the main text.

A first branch had a ω of 0.86 and represented the evolution of the common ancestor of *Ungulata *to the common ancestor of *Artiodactyla *(node 30–29, Figure 2, additional file 5: ω along tree branches). This ratio was based on 34 non-synonymous exchanges between both ancestral sequences of which 28 are within LRR, three within the transmembrane domain and one within the TIR domain (additional file 6: AA substitutions along discussed branches). Twenty-five of these AA sites displayed high ω and were assigned to AA site class 3 (potentially positive selected) in the complete dataset, but only five were assigned to site class 3 in the ruminant set of sequences. All remaining 20 AA sites assigned to class 3 in all mammals studied were assigned to class 2 in the ruminants.

A further branch separated bovine ruminants (*Bovinae*, node 26) from small ruminants, *Giraffa camelopardalis *and all non-ruminants (node 27). It had a ω of 1.22 and was based on ten exchanges affecting AAs, of which seven were located within LRRs, one in the transmembrane domain, and one in the TIR domain (additional file 6: AA substitutions along discussed branches). Five of these 10 affected sites were classified as class 3 (potentially positively-selected) in the complete dataset. Using the ruminant dataset, eight were classified as class 3 AA sites, of which four had intermediate ω (class 2) in the complete dataset. Only one AA site was assigned to class 3 in the complete dataset, but to class 2 in the ruminant dataset. In contrast to all other branches, this bovine branch was based on exchanges affecting AA sites which were specifically under positive selection in ruminants, but not in the complete mammalian dataset.

The third branch, separating *Ovis aries *and *Capra ibex *(node 43) from the other ruminants (node 41) had a ω of 0.95 and affected six AAs, all of which were located within the first 326 AAs of the extracellular domain (additional file 6: AA substitutions along discussed branches). Four of these AA sites were assigned to AA site class 3 in the complete dataset and three in the set of sequences limited to ruminants. One AA site assigned to class 3 overall was assigned to class 2 in the ruminant dataset.

### Branches based on abundant or unevenly distributed exchanges

A further three branches had average ω, but displayed a noteworthy number of substitutions or a remarkable bias of distribution of the exchanges over the protein sequence (additional file 6: AA substitutions along discussed branches).

The first of these branches connected the common ancestor of *Artiodactyla *(node 29) with the common ancestor of the ruminants (node 28). It had a ω of 0.562 and consisted of 80 exchanges, of which 49 were assigned to class 3 in the complete dataset, but only eight to class 3 in the ruminant set of sequences. Forty-one AA sites assigned to class 3 in the complete dataset were assigned to class 2 in the ruminant dataset.

The second branch separated the common ancestor of ruminants (node 28) from the *Bovidae *(node 27). It had a ω of 0.248 and contained seven exchanges, of which five were assigned to class 3 in the complete dataset and four to class 3 in the ruminant dataset. One changed from class 3 in the complete dataset to class 2 in the ruminant dataset. All these exchanges were located in the N-terminal half of the protein between AA101–AA453.

The third branch separating the common ancestor of the *Bovidae *(27) from small ruminants (node 40) had a ratio of 0.511 based on 11 exchanges. In contrast to most of the TLR2 AA sites in ruminants, most of these exchanges were classified as probably positively-selected (class 3) in both sequence sets, even those located in the normally highly conserved TIR domain. Three AA sites changed from a class 3 assignment in the complete dataset to a class 2 assignment in ruminants and one from a class 2 assignment in the complete dataset to a class 3 assignment in the ruminant dataset. All of these exchanges were located in the C-terminal half of the protein between AA508–AA773, with more than half of them located in the highly-conserved TIR domain.

### Polymorphism in bovine TLR2 and detection of polymorphic AA sites under positive selection

Sequencing of DNA pools containing amplified TLR2 coding regions of 10 cattle breeds revealed 29 polymorphic sites in the nucleotide sequence. One of these has been reported previously (silent exchange RefSNP# RS41830058 at nt 1707). They result in 20 yet unreported polymorphic AA sites in the bovine TLR2 (Table 2). Sixteen of them were located in LRR domains, one in the transmembrane domain and three in the TIR domain. Ten involved exchanges between AAs which occurred rarely on a genome scale with a Blosum62 score of 0 or lower, indicating that these exchanges were likely to have functional effects. Nine of the non-synonymous SNPs were assigned to the site class likely to be positively-selected when comparing the sequences of all analysed mammals. Only six of these fitted this category when comparing sequences within ruminants. All these six exchanges in ruminants have a Blosum62 score of 0 or lower, again indicating a higher likelihood of functional effects.

**Table 2:** Polymorphism in cattle

Nucleotide	189	202	455	467	631	681	914	925	978	989
Amino acid	63	68	152	156	211	227	305	309	326	330
**Domain**	LRR1	LRR1	LRR5	LRR5	LRR7	LRR8	LRR10	LRR11	LRR11	LRR11
**Blosum62**	2	0	1	-3	3	0	-2	1	0	1

**P (all)**	0.922	0.735	0.22	0.005	0.942	0	1	0	0.982	0.018
**Class (all)**	3	3	2	2	3	1	3	1	3	2
**Class (ruminants)**	2	3	2	3	2	3	3	2	3	2

**Codon_wildtype**	ga**g**	**g**gc	c**g**g	g**t**a	**g**tt	tt**a**	c**a**c	**g**tg	ca**t**	a**g**t
**Codon_mutant**	ga**t**	**a**gc	c**a**g	g**g**a	**a**at	tt**t**	c**c**c	**a**tg	ca**a**	a**a**t
**AA_wildtype**	E	G	R	V	V	L	H	V	H	S
**AA_mutant**	D	S	Q	G	I	F	P	M	Q	N

**dbSNP_ID (ss#)**	95214853	95214854	95214855	95214856	95214857	95214858	95214858	95214859	95214861	95214862

**AA**	t	g	g	t	a	t	a	g	t	g
**FB**	t	g	g	t	g/a	t	a	g	t	a
**BP**	t	g	g	g	g/a	t	a	g	t	g
**GA**	g/t	g/a	g	t	g	t	a	g	t	g
**FV**	t	g	g/a	t	g/a	t	a	g/a	t	g
**HF**	t	g	g	t	a	t	a	g	t	g
**LI**	t	g	g	t	g/a	t	a	g	t	g

**NE**	g/t	g/a	g	t	g	a	a	g	a/t	g
**SW**	g	a	g	t	g	a	c	g	a	g
**BH**	g	g/a	g	t	g	a/t	a	g	a	g

										

**Nucleotide**	**1010**	**1214**	**1250**	**1457**	**1504**	**1688**	**1814**	**1962**	**1995**	**2149**
**Amino acid**	**337**	**405**	**417**	**486**	**502**	**563**	**605**	**654**	**665**	**717**

**Domain**	LRR11	LRR14	LRR15	LRR18	LRR19	LRRCT	MEM	TIR	TIR	TIR
**Blosum62**	2	-1	1	2	1	0	-1	-2	0	2

**P (all)**	0.972	0	0.352	0	0.998	0.776	0.578	0	0.011	0
**Class (all)**	3	1	2	1	3	3	3	1	2	1
**Class (ruminants)**	2	2	2	2	2	3	2	2	2	2

**Codon_wildtype**	a**a**a	a**c**g	a**a**c	a**g**a	**t**ca	c**g**c	a**c**g	tg**g**	ca**c**	**c**tg
**Codon_mutant**	a**g**a	a**t**g	a**g**c	a**a**a	**g**ca	c**a**c	a**t**g	tg**t**	ca**g**	**a**tg
**AA_wildtype**	K	T	N	R	S	R	T	W	H	L
**AA_mutant**	R	M	S	K	A	H	M	C	Q	M

**dbSNP_ID (ss#)**	95214863	95214864	95214865	95214867	95214868	95214869	95214873	95214874	95214875	95214878

**AA**	g	c	a	g	t	g/a	c	g	c	c
**FB**	g	c	a	g	t	g	c	g	c	c
**BP**	g	c	a	g	t	g	c	g	c	c
**GA**	g/a	c	a	g	t	g/a	c	g	c	c
**FV**	g	c	a	g	t	g	c	g	c	c
**HF**	g	c	a	g	t	g	c	g/t	c	a/c
**LI**	g	c	a	g/a	t	g	c	g	c	c

**NE**	a	c	g/a	g	g/t	g/a	c/t	g	g/c	c
**SW**	a	t	g	g	g	a	c/t	g	g	c
**BH**	a	c	g	g	t	a	c	g	g	c

Nucleotide: position of the polymorphism in the nucleotide sequence, Amino acid: affected amino acid site, Domain: domain in which the polymorphic site is located, LRRCT: Leucine Rich Repeat C-terminal domain, MEM: transmembrane domain, Blosum62: Blosum62 matrix score of the particular AA exchange, P (all): probablitiy of class assignment in the complete dataset, Class (all): class assignment in the complete dataset (1: purifying selection, 2: neutral, 3: positive selection), Class (ruminants): class assignment in the ruminant subset, dbSNP_ID (ss#): accession number in dbSNP database, AA: Aberdeen Angus, FB: Fighting Bull, BP: Black Pied Gene Reserve, GA: German Angus, FV: Fleckvieh, HF: Holstein Friesian, LI: Limousin, NE: Nelore, SW: Sahiwal, BH: Brahman. The last three breeds are *Bos indicus *breeds.

Four of the 20 non-synonymous substitutions were fixed in all breeds analysed, two of them (AA305 and AA405) with Sahiwal breed-specific AAs, one with a fixed exchange which was present only in the Spanish Fighting Bull (AA330) and a fourth one with a fixed exchange present only in the German Black Pied (AA156). Three mutations at positions 326, 417 and 665 were specific to *Bos indicus *with all taurine breeds fixed for the wildtype.

## Discussion

### Patterns of selective constraint on TLR2 ligand binding, dimerisation & AAs involved in signalling

The evolution of the mammalian *TLR2 *gene has been shaped by positive and purifying selection (Figure 1). Sequence variation at AA sites responsible for pattern recognition (PAMP ligands) will have enabled a faster adaptation to new pathogens as they are encountered in different geographical locations or as they newly emerge in a habitat. This has clear selective advantages. Such advantageous substitutions have spread faster in the populations analysed than random substitutions. They appear concentrated in the extracellular region of TLR2, particularly in the LRRs 8–10 in which residues responsible for ligand binding have recently been reported [9]. In contrast AAs responsible for functions like heterodimerisation or signalling were more conserved, presumably because the interacting molecules require conserved AA binding sites. Amino acids essential for TLR2 dimerisation with TLR1 in human and mouse have been identified in the LRRs 11, 12, 13, 14 and the TIR domain [9,12,13] (additional file 7: Functional relevant TLR2 sites identified in former studies in human or mouse). Our results show that, with the exception of LRR11, all these regions were under strong purifying selection whereas LRR12 and 13 were amongst the most conserved regions of the extracellular domain. The TIR domain was the most conserved domain of the protein, which is known to be conserved even among TLR paralogs [12]. In human and mouse TLR2, Jin *et al. *[9] identified both ligand binding and TLR1 heterodimerisation sites within LRR11 (additional file 7: Functional relevant TLR2 sites identified in former studies in human or mouse). According to our assumptions, this would lead to opposed selective pressures on different AA sites within the same domain. This was in fact observed and resulted in an overall average ω for this LRR in bovine TLR2.

We have assumed in this study that many of the TLR domains have similar functions in all species and that the data from human and mouse TLRs regarding dimerisation and ligand binding sites can in most cases be extrapolated to other mammalian species. Having said this, we are aware that functional changes can occur during evolution that could lead to taxon-specific selective pressures.

### Taxon-specific signatures of selection

Traces of selection detected in large, diverse mammalian sequence sets alone do not necessarily reflect substitutions which are of current functional relevance for any one species. Such patterns could also be the result of ancient selection events which triggered functional changes which afterwards became fixed or gradually lost their importance. Therefore we analysed in addition a separate set of ruminant sequences. Differences between the complete mammalian dataset and the ruminant dataset should reflect functional changes during evolution and could help to highlight those AA sites which may be of current functional importance in cattle (additional file 3: Comparative plot of the average ω of a 20 AA sliding window along the protein sequence).

During the emergence of the *Artiodactyla *there was a strong positive selection on the *TLR2 *gene. However, in the subsequent period of ruminant evolution, the majority of potentially-positive selected class 3 AA sites seen in mammals seem to have returned to a nearly neutral evolution within ruminants. One reason for this is that the branches within ruminants are much shorter than in the extended species phylogenetic tree. The power of site classification has therefore limitations and has resulted in much less class 1 and 3 assignments. However, the absolute number of ω in ruminants nearly always pointed to less selective constraint in either direction. This has resulted in a lower variance of ω in ruminants than in mammals generally (additional file 3: Comparative plot of the average ω of a 20 AA sliding window along the protein sequence, F-test, *P = *0.03).

Many TLR2 LRR motifs which contain sites known to be responsible for ligand binding in human or mouse and display high ω in the entire mammalian dataset were considerably less positively-selected in ruminants (e.g. LRR9, 10, 11). In a remarkable contrast, most of those domains which are crucial for dimerisation and under strong purifying selection in the complete mammalian dataset are *less *conserved in ruminants (LRR12, 13 and TIR).

Therefore we speculate that this might reflect functional changes of these sites during evolution, releasing these AAs from strong selective pressure in ruminants. Differences between species in ligand recognition are known for several TLRs [30,31] and can result in species-specific immune responses to certain pathogens.

### Exchanges along branches

The above speculation is also supported by the exchanges along the branches of a phylogenetic tree. During the emergence of the *Artiodactyla *there was a strong positive selection on the *TLR2 *gene, whereas in the subsequent period involving the spread of ruminants the majority of initially positive selected class 3 sites on TLR2 return to a nearly neutral evolution characterized by average ω along the branches (additional file 5: ω along tree branches).

However, there are two exceptions to this general pattern for ruminants as a whole: the separation of small and large ruminants and the evolution of ovine TLR2 in ibex and sheep. The TLR2 of the common ancestor of *Bovidae *(node 27) separated into two branches corresponding to small (node 40) and large (node 26) ruminants. The first branch (nodes 27, 40) is based on an extraordinary concentration of AA exchanges in the TIR domain on AA sites which were driven by positive selection (additional file 6: AA substitutions along discussed branches) and occurred at positions near to AA sites which were reported to be responsible for heterodimerisation with TLR1 in human and mouse [9]. Gene conversion events between TLR1 and 6 [22] could trigger such positive selection on AA sites which are normally under strong purifying selection, resulting in high ω for such AA sites in the otherwise highly conserved domain. Similarly, most exchanges of the second branch (nodes 27, 26) leading to large bovine ruminants (*Bovinae*) were under positive selection in both the general mammalian and ruminant datasets (additional file 6: AA substitutions along discussed branches). In fact this was the only branch in which more altered sites were under positive selection in ruminants than in mammals, including four AA sites which were classified as class 2 in mammals, but as class 3 in ruminants. It is possible that these exchanges were caused by adaptive selection and resulted in bovine specific functional changes.

Contrary to the generally observed phylogeny of TLR2 in the different species, the TLR2 of *Capra ibex *and *Ovis aries *cluster closely together but relatively distant from *Capra hircus*. The branch separating both species from the other small ruminants (nodes 41–43) is formed by exchanges which are all concentrated in a region within the first 326 AA of the protein, affecting LRR domains which are thought to be responsible for ligand binding. One of the exchanges (AA326) affected a position known to be crucial for lipopeptide detection in human TLR2 (and that is polymorphic in cattle – see below), and two further substitutions were located near these sites [9]. High ω were probably the product of adaptation to specific pathogens which is known to trigger diversifying selection [25]. The sequences might reflect specific adaptation to certain microbial/pathogen environments. In this case, a high variation within species would be expected in line with the different environments in which these species evolve. This would predict polymorphisms in common in several closely related species and possibly appear fixed in certain environments. The *Capra hircus *sequence originated from an Indian sample, while the *Ovis aries *and *Capra ibex *individuals were both from Europe. The different clustering might therefore reflect the geographic origin rather than the differences between species.

### Determination of polymorphic sites likely to have functional effects

The relative genetic disparity between *Bos indicus *and *Bos taurus *is well established [32] and this genetic distance would explain some of the TLR2 polymorphisms observed between these two subspecies. However, the difference would not just be defined by random events over the time they diverged, but also by different microbial environments that the animals evolve within. The *Bos indicus *breeds analysed herein originated in tropical or subtropical climates where the pathogen population is generally different to that encountered by the *Bos taurus *breeds sampled which all inhabited Europe. Consequently, there will be different selective pressures acting on the TLR and other immune related genes in these subspecies, which would result in sites being differentially fixed in their TLR genes.

Functionally-important AAs in the ligand-binding domains of the TLRs would be shaped by selection. Conserved AA sites are protected by purifying selection and therefore normally fixed. Sporadic mutations at such sites are likely to disappear very quickly from any of the population they have occurred in. This is probably the case for the substitutions found at positions 309, 486, 654 and 717 (Table 2). All four exchanges were polymorphic within a single breed and it can be assumed that these are random mutations, possibly with no functional effect and low frequency. Neutral mutations (class 2) are expected to have no functional relevance. They might have appeared randomly and spread or disappeared driven by genetic drift rather than by selection. In our dataset, such exchanges (positions 152, 330, 417, 665) appeared mostly in only one breed and at low frequency. In such circumstances functional relevance is unlikely. In contrast, AA sites that are shaped by positive selection are expected to spread quickly in populations in which their occurrence has positive functional effects.

Sites which are shaped by positive selection in the complete mammalian dataset, but not in the ruminant dataset might have lost their functional significance during evolution. Five polymorphic sites fell into this category (AA63, AA211, AA337, AA502, AA605) and all but position 605 had a relatively common AA substitution (Blosum62 > 0), which indicates that functional effects in cattle are not very likely.

In contrast, AA sites which are positively selected in ruminants, but not in the complete dataset are candidates for ruminant-specific TLR2 functions (156, 227). This is likely particularly if such sites are differentially fixed in breeds originating from or evolving in different geographical (hence microbial) environments (Table 2). The substitution Leu227Phe was fixed for Phe in all *Bos taurus *breeds analysed whereas the wildtype Leu occured only in *Bos indicus*. This substitution had a low Blosum62 score which suggests that the establishment of a Leu-Phe replacement in any given population is a scarce event and therefore is more likely to affect function. This pattern could be explained by a selective advantage of Phe in a temperate environment in which the sampled *Bos taurus *breed originated and thus this polymorphism is of interest for future functional study.

Functional effects are also possible from polymorphisms at AA sites which are positively selected in both ruminants and mammals generally (68, 305, 326, 563). These AA sites are most likely to be of functional importance if they are differentially fixed in breeds evolving in different microbial environments. For example, at positions 326 and 563 of TLR2 the mutations were fixed in *Bos indicus *breeds sharing common environments (Table 2).

Functional consequences seem likely for His305Pro with a *P*-value for class 3 assignment of 1 and a seldom AA exchange (Blosum62 score of -2). This AA was subject to a frequent change during evolution (additional file 6: AA substitutions along discussed branches). In all other ruminants only His and Tyr occurred at this site. Pro appears to be a new mutation which possibly occurred recently in *Bos indicus *and was only observed in the Sahiwal sample. A higher number of Sahiwal individuals is needed to estimate the frequency of occurrence of this allele and whether a positive functional effect is predicted.

The His326Gln exchange in TLR2 takes place within the ligand-binding region of TLR2 [9], but where the human TLR2 positional homologue is a Tyr. Tyr and Gln are uncharged, in contrast to His which is positively charged. During the phylogeny of TLR2 this position has changed several times, including back mutations (branch 41–43) which suggest adaptive positive selection (additional file 6: AA substitutions along discussed branches). The fixation of the wildtype in *Bos taurus *and of the mutant in two of three *Bos indicus *breeds indicates that possible adaptive selection might depend on the environment the animals are held in. This exchange is therefore likely to have functional consequences for the ligand binding ability of bovine TLR2.

Similarly, the occurrence of Arg563His in different breeds could be explained by adaptive selection. However, the site appears to be unchanged in nearly all ruminants (Arg), while all non-ruminants are fixed with Leu at this position, pointing to a ruminant specificity with a high level of conservation. The *P*-values for the class 3 assignments for this are not robust and raise the issue that this could be a neutrally evolving polymorphism which spreads due to genetic drift.

## Conclusion

Within bovine TLR2, we have identified that selective constraint has acted differentially on sites associated with domains with distinct functions. Amino acids responsible for TLR-PAMP ligand binding have been shaped for the most part by positive selection, while sites linked to TLR heterodimerisation are conserved. This suggests that adaptive selection shapes ligand binding sites to improve the recognition of different relevant pathogens and that AA at such sites reflect microbial environments. Polymorphism affecting these AAs is therefore likely to result in functional consequences.

However, sites can lose or gain functional importance during evolution. This seems to be of particular importance in ruminants as reflected in a change of selective constraint on certain AAs. This could have implications for the way the ruminant innate immune system works and should be further investigated using comparative functional studies.

Polymorphism in bovine TLR2 occurs mainly between breeds and is probably associated with different geographic and therefore microbial environments. This could be investigated further by population genetic studies which would link the occurrence of certain variants with certain pathogen environments.

Polymorphic sites likely to be functionally relevant in bovine TLR2 were predicted by analysis of: selective constraint, molecular evolution, extrapolating information about TLR domain functions from human and mouse to cattle and estimating the effect of the AA exchanges observed. Such sites have been identified at positions 227, 305 and 326 of the TLR2 predicted polypeptide and affect AAs or domains known to be involved in the recognition of PAMP ligands by TLR2.

A final proof of their functional relevance requires further studies to determine their effect on the immune response after stimulation with relevant ligands and/or their association with immune related traits in animals. This would facilitate the identification of particular disease susceptibility/resistance in cattle and will provide a valuable tool for the breeding industry to improve genetic resistance against a range of pathogens.

## Methods

### Sample collection

DNA from 90 individuals belonging to 7 *Bos taurus *and 3 *Bos indicus *breeds of different geographic origin were collected. In addition DNA and tissue samples of 7 ruminant species were sampled and DNA extracted using standard procedures (Qiagen kit) (Table 3).

**Table 3:** Collected DNA samples of ruminant species and cattle breeds

Species	Breed	Origin	Sampled in	N	Acc#
*Bos taurus*	German Angus	Germany	Germany	12	
*Bos taurus*	Aberdeen Angus	UK	UK	10	
*Bos taurus*	Holstein Friesian	UK	UK	10	
*Bos taurus*	German Black Pied	Germany	Germany	8	
*Bos taurus*	Fighting Bull	Spain	Spain	12	
*Bos taurus*	Limousin	France	France	2	
*Bos taurus*	German Simmental	Germany	Germany	11	
*Bos indicus*	Brahman	USA	Paraguay	12	
*Bos indicus*	Nelore	Brazil	Paraguay	12	
*Bos indicus*	Sahiwal	Pakistan	UK	1	
*Capra ibex*	-	Switzerland	Switzerland	1	EU580540
*G. camelopardalis*	-	South Africa	South Africa	1	EU580542
*A. marsupialis*	-	South Africa	South Africa	1	EU580538
*D. dorcas phillipsi*	-	South Africa	South Africa	1	EU580541
*Bison bison*	-	USA	USA	1	EU580539
*Ovis aries*	Suffolk	UK	UK	1	EU580543

N: sample size, Acc#: Genbank accession number,*G. camelopardalis: Giraffa camelopardalis*, *A. marsupialis: Antidorcas marsupialis*, *D. dorcas phillipsi: Damaliscus dorcas phillipsi*

### Primer design

Bovine primers were designed using the bovine sequence assembly Btau_3.1 (NW_929136.1) using Primer3 software [33]. One amplification primer pair was designed to cover the complete coding sequence in one fragment of 2467 bp. Twelve sequencing primers were designed on both strands with an average distance of 500 bp, whereas the most outlying primers were designed as nested primers to avoid sequencing background of unspecific PCR products (Table 4). Ruminant species were first amplified and sequenced using bovine primers. Gaps in the sequences due to failed sequencing reactions were covered by redesigning primers based on the successful reads (Table 4).

**Table 4:** Used primers

Primer_ID	Sequence
*TLR2.0_600_5'*	TAAGCCATGATGTCAAACACAG
*TLR2.0_600_3'*	TTTCCTACTTTTAGGGTCCGC
*TLR2.1350_1950_5'*	AAACTTGTCAGTGGCCAGG
*TLR2.1350_1950_3'*	TGGAAACGGTGACACAGC
*TLR2.1866_2466_5'*	TGCTGTGTCACCGTTTCC
*TLR2.1866_2466_3'*	GGATCCTAGGACCTTATTGCAG
*TLR2.450_1050_5'*	CAAAACACTTGGGGAAACATC
*TLR2.450_1050_3'*	TCCGTATTGTTAACGTCTCCAC
*TLR2.900_1500_5'*	GACTGTACCCATGATGGAATTG
*TLR2.900_1500_3'*	TAAAATTTCCAGGGTCTGGG
*TLR2.end_f*	GCCATGATGTCAAACACAGTC
*TLR2.end_r*	CACCACCAGACCAAGACTGA
*TLR2_f*	TGGAATTAAGCCATGATGTCAA
*TLR2_r*	GACCACCACCAGACCAAGAC
*Bison_contig1_343_r*	GCAAGTGGATCATCGACAAC
*Blesbuck_contig1_347_r*	GACCTGAACCAGGAGGATGA
*Blesbuck_contig1_721_r*	TTCACTGATGGATGCTTCTGA
*Blesbuck_contig1_1047_r*	AAACAAGGAACCAGGAAAACC
*Ibex_contig1_591_f*	TGAGGAGCTTGAGATCAGTGC
*Ibex_contig1_2443_r*	CCAAGACTGACCCTTAACGAA
*Giraffe_contig1__268_r*	TTTGTGAAGAGCGAGTGGTG
*Giraffe_contig1__980_r*	CGATTCATTTTCTTTGATTTTGC
*Giraffe_contig1__1229_r*	GCCCTTCCTTCAAACCTTG
*Impala_contig1_207.r*	TGCACTGATCTCAAGCTCCTC
*Impala contig1_466.f*	TGATGAAAGTTTTGTTGAAGTTG
*Impala contig2_150.r*	AAACAAGGAACCAGGAAAACC
*Impala contig2_705.r*	CAGGAGCAAATGAAGTTGTTG
*Impala contig2_700.f*	GTGGCAACAACTTCATTTGC
*Impala contig3_7.f*	GAGGGAAGCCCAGGAAG
*Impala contig3_26.r*	AGCCTTCCTGGGCTTCC
*Impala contig3_473.f*	TTTGAGAGCTGCAATACGG
*Impala_contig4_79.f*	GGGACTGAACCAGGAGGATG
*Impala_contig4_312.r*	AACTGGTGTCTGCGATGG
*Sheep_contig1_600.r*	GAAAAGTCAGTCCAGTGAAATCC
*Sheep_contig1_650.f*	TGCAGTTGTATGTGCCAAAG
*Sheep_contig2_167.r*	TTTTAACTTTGCCTGTGAGTGG
*Sheep_contig2_1435.f*	CATGAACACCAGGACCTACC
*Springbok_contig1_135.r*	GTCTTGTGACCCAACTGGTG
*Springbok_contig1_1733.r*	GTCACAGCGGTAGCCATCTG
*Springbok_contig1_1734.f*	ATGGCTACCGCTGTGACG
*Springbok_contig1_2269.f*	GAGCCCATTGACAAGAAGG

### PCR conditions

PCR was performed using a proof-reading DNA polymerase (DyNAzyme™ EXT DNA Polymerase, New England Biolabs). 40 ng of DNA was amplified in 50 μl volumes, using 1.5 mM MgCl_2_, 0.25 μM each primer, 200 μM each dNTP and 1.5 U DNAzyme EXT DNA Polymerase.

### Template preparation and sequencing

Amplified DNA was cleaned using Multiscreen filter plates (Millipore), quantified and the cattle amplicons breed-wise pooled. Sequencing reactions were conducted in 10 μl volumes containing 60 ng template, 0.5 μl ABI BigDye Terminator Mix V3.1, 1 μl Dilution Buffer, 1 μl primer and 0.5 μl DMSO. Cycle conditions were 3 min at 96°C, followed by 30 cycles of 30 s at 96°C, 20 s at 50°C and 4 min at 60°C. Sequencing reactions were consequently washed with 70% Ethanol and sequenced on an ABI 3730 automated sequences (Applied Biosystems).

### Public sequences used

In addition to the sequenced species publicly available coding sequences of the several mammalian species were used: *Mus musculus *(NM_011905.2), *Pan troglodytes *(XM_001155304.1), *Rattus norvegicus *(NM_198769.2), *Boselaphus tragocamelus *(DQ286731.1), *Bubalus bubalis *(DQ288130.1), *Oryctolagus cuniculus *(NM_001082781.1), *Macaca fascicularis *(AY045573.1), *Macaca mulatta *(XM_001087830.1), *Homo sapiens *(NM_003264.3), *Equus caballus *(AY429602.1), *Cricetulus griseus *(AF113614.1), *Capra hircus *(DQ872435.1), *Canis familiaris *(NM_001005264.1), *Sus scrofa *(AB208696.2), *Monodelphis domestica *(ENSMODG00000024954).

### Sequence analysis

Sequences were processed, analysed and assembled using the programs pregap and gap of the Staden sequence analysis package [34]. Consensus sequences were created and all inconsistencies and polymorphisms examined by eye. Created sequences were uploaded to the NCBI GenBank, accession numbers are indicated in Table 3.

Sequences were aligned using ClustalW and translated. The domain structure of the extra-cellular domains was deducted from the TLR2 LRR structure published by Matsushima *et al. *[11]. The transmembrane and intracellular domains were predicted using information provided in Xu *et al. *[12] and retrieved from the SMART online database at [35] (domain structure embedded in additional file 2: Class assignment of AA sites).

### Entropy plots

Sequence conservation along alignments was visualized using an entropy plot as implemented into Bioedit [36]. It is a measure of the lack of predictability for an alignment position. A score of 0 stands for complete conservation (completely predictable), high values for high diversity between sequences. For a comparison between groups values were corrected for branch-depth (overall conservation) by normalizing the values to an identical average in all alignments. The resulting frequencies were used to calculate a sliding average value for a 50 AA window, which was plotted in a chart (see additional file 1: Comparative plot of the average corrected entropy of a 50 AA sliding window along the protein sequence).

### Evaluation of AA exchanges

The probablility of functional consequences of non-synonymous substitutions depends on the AAs involved. To evaluate the significance of substitutions we used the Blosum62 matrix [37]. This attributes a score based on the observed frequencies of such occurences in alignments of related proteins. Frequently observed substitutions are likely to be functional neutral and receive positive scores, while seldom observed substitutions are seen to be more likely to cause functional disturbance and are given negative scores.

AA sites found to be polymorphic in cattle were compared to the consensus of ruminant species. The consensus AA was assumed to be the bovine wildtype.

### Phylogenetic Analysis using Maximum Likelihood

An analysis of functional constraint and molecular evolution of the *TLR2 *gene was conducted using Maximum Likelihood based algorithms included into PAML [38].

*Along sites: *ratios between synonymous and non-synonymous substitutions (ω) along AA sites were estimated with the program codeml of the PAML package, using Model M3, with K = 3 AA site classes as in Yang *et al. *[24]. The algorithm estimates for each AA site an assignment probability to three possible site classes with low ω (purifying selection), intermediate ω (neutral) or high ω (positively selected) and allocates the site to the class with highest probability.

Ratios along sites were estimated assuming only one ω over branches (model = 0). Selective constraint on the different protein domains was visualized by an Excel chart with a sliding window displaying the average ω of the surrounding 20 AAs (Figure 1).

*Group specific ω along AA sites: *in addition ω were estimated along AA sites as described above, but with a dataset limited to ruminant sequences. Class allocations were compared between the ruminant and the complete dataset for each AA. Sites allocated to opposite classes (purifying *versus *positive selection) in ruminants were evaluated as shaped by a taxon specific selective pressure.

Variance of ω was estimated and tested using an F-test as established in Excel. The hypothesis 1 that the variation of ω among sites is identical between ruminants and all species is tested against the hypothesis that ω differs between both groups.

*Along branches: *nevertheless ω along branches were estimated using the free-ratio model, allowing a different ratio along each branch. In this model the same ω along sites was assumed (M0). Using this model also ancestral sequences were reconstructed.

### Phylogenetic trees

Phylogenetic trees from an alignment of DNA sequences were constructed by calculating a distance matrix using the F84 model in DNAdist version 3.5 c, tree files were calculated using NEIGHBOR [39]. Generated tree files were then used as input file for PAML. Phylogenetic trees based only on synonymous or non-synonymous exchanges were generated by PAML. Trees were visualized by treeview [40].

## Abbreviations

AA: Amino acid; PAMP: Pathogen associated molecular pattern; TLR: Toll-like Receptor; VS: Variable Segment of Leucine Rich Repeat; HCS: Highly conserved segment of Leucine Rich Repeat; LRR: Leucine Rich Repeat. ω: dN/dSratio; PAML: Phylogenetic Analysis by Maximum Likelihood; TIR: Toll/interleukin-1 receptor; Phe: Phenylalanine; Arg: Arginine; Leu: Leucine; Tyr:Tyrosine; Glu: Glutamic acid; Gln: Glutamine; His: Histidine.

## Authors' contributions

OJ organized the sample collection, amplified, pooled and sequenced the samples, analysed sequences, conducted the phylogenetic and PAML analysis and drafted the manuscript. DW contributed to the prediction of functional effects and helped with the drafting of the manuscript. JC sampled and sequenced sheep cds. DH coordinated the project and helped with the drafting of the manuscript. EJG designed and supervised the study and helped with the drafting of the manuscript.

## Supplementary Material

Additional file 1 **Comparative plot of the average corrected entropy of a 50 AA sliding window along the protein sequence**. The plot is based on the complete dataset of 22 mammalian sequences (green line) and a subset limited on ruminants (blue line). Click here for file

Additional file 2 **Class assignment of AA sites**. The positions (AA Pos) are numbered according to the cattle AA sequence. The AA at each position in cattle, p-values for class assignment (p1: purifying selection, p2: neutral, p3: positive selection), classes & ω for all mammals and ruminants (all class/w, ruminants w/class) are indicated. The domain structure is highlighted by coloured background beginning at AA54 and d_N_/d_S _ratios of the domains in the complete and the limited ruminant dataset are indicated in comment boxes. The domain structure was deducted from Matsushima *et al. *[11] if not noted otherwise. Sites marked with * do not exist in cattle, but in other species of the alignment. Click here for file

Additional file 3 **Comparative plot of the average ω of a 20 AA sliding window along the protein sequence**. The plot is based on the complete dataset of 22 mammalian sequences (green line) and a subset limited on ruminants (purple line). Click here for file

Additional file 4 **Unrooted radial phylogenetic trees of the analysed TLR2 sequences**. The d_N _tree displays the relationship between the sequences using non-synonymous exchanges only, the d_S _tree is limited on synonymous exchanges. The given scale represents a distance of 0.1 nucleotide exchanges per codon. Click here for file

Additional file 5 **ω along tree branches**. Node numbers refer to Figure 2. Click here for file

Additional file 6 **AA substitutions along discussed branches**. The indicated positions (Pos cattle) refer to the cattle TLR2 protein sequence. The amino acid present at the corresponding position of the ancestral sequences (AA 1./2. node) and the involved taxa are indicated. Node numbers refer to Figure 2. Affected domains (domain) and d_N_/d_S _ratios of the site in the complete and the ruminant dataset (w all/rum) are given. The site class assignment in the complete and ruminant dataset (class all/rum) and the class differences (class diff) between both datasets are indicated. Sites which are under stronger positive selection in ruminants than in the complete dataset are highlighted by red background. Click here for file

Additional file 7 **Functional relevant TLR2 sites identified in former studies in human or mouse**. The amino acid at the corresponding position in cattle, and the function of this particular site in the indicated species is given. Click here for file
