# Supplementary figures and images for: Molecular evolution of bovine Toll-like receptor 2 suggests substitutions of functional relevance
Source: BMC Evol Biol. 2008 Oct 20;8:288. doi: 10.1186/1471-2148-8-288 (PMC2588590; doi:10.1186/1471-2148-8-288)

## Slide 1
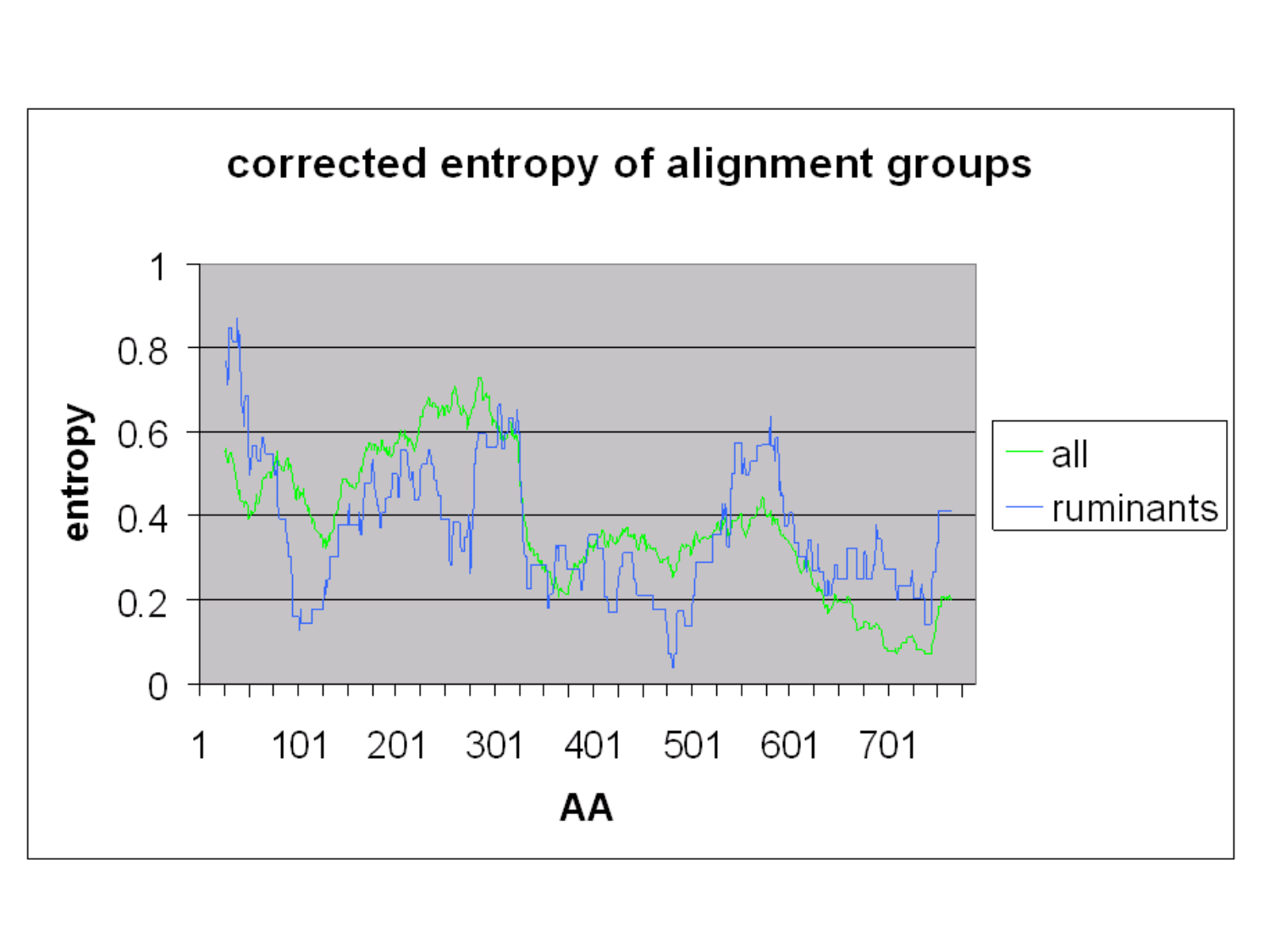

Supplement: Additional file 1 — Comparative plot of the average corrected entropy of a 50 AA sliding window along the protein sequence. The plot is based on the complete dataset of 22 mammalian sequences (green line) and a subset limited on ruminants (blue line). [file 1471-2148-8-288-S1.ppt]

## Slide 1
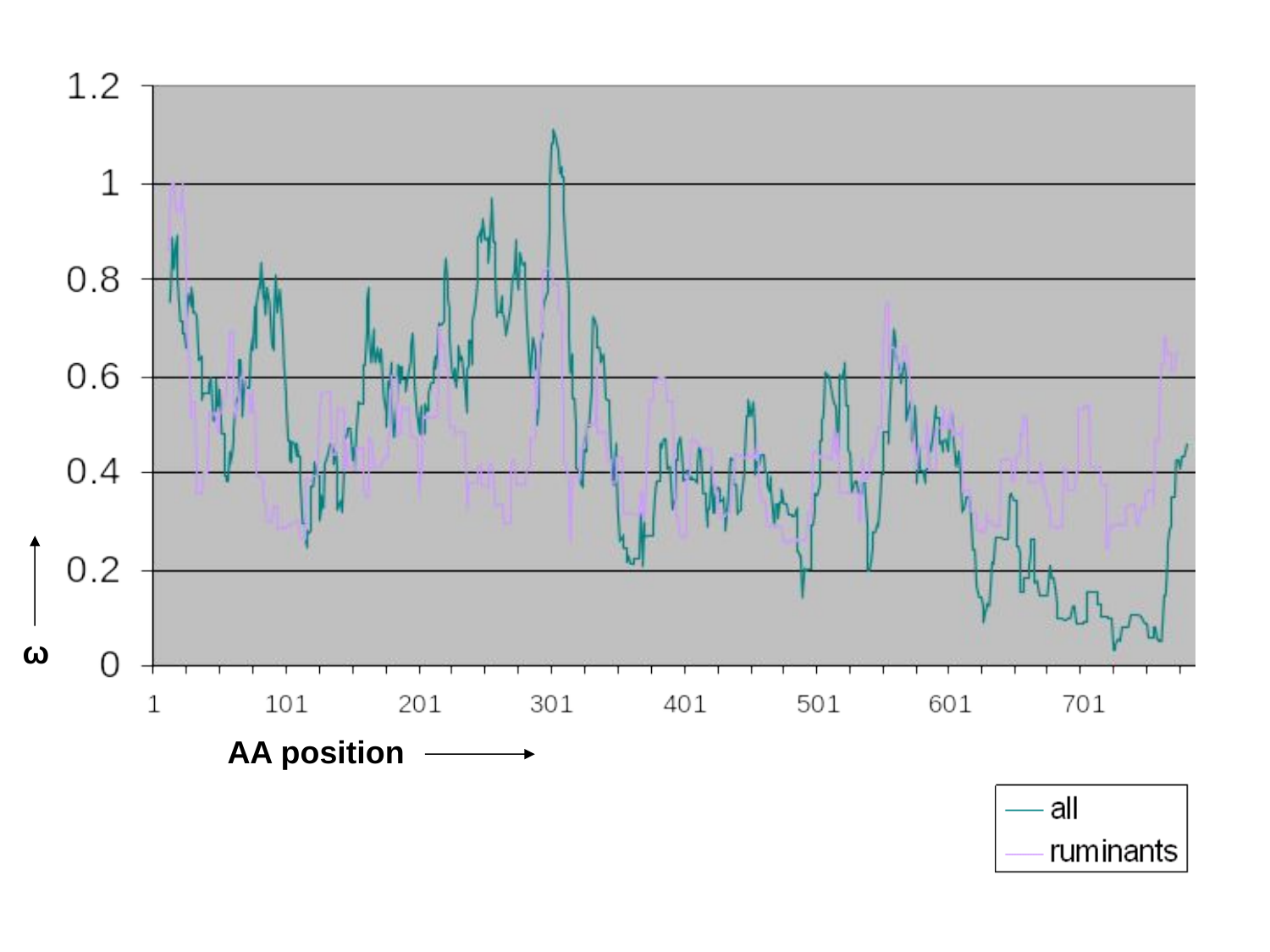

ω
AA position

Supplement: Additional file 3 — Comparative plot of the average ω of a 20 AA sliding window along the protein sequence. The plot is based on the complete dataset of 22 mammalian sequences (green line) and a subset limited on ruminants (purple line). [file 1471-2148-8-288-S3.ppt]

## Slide 1
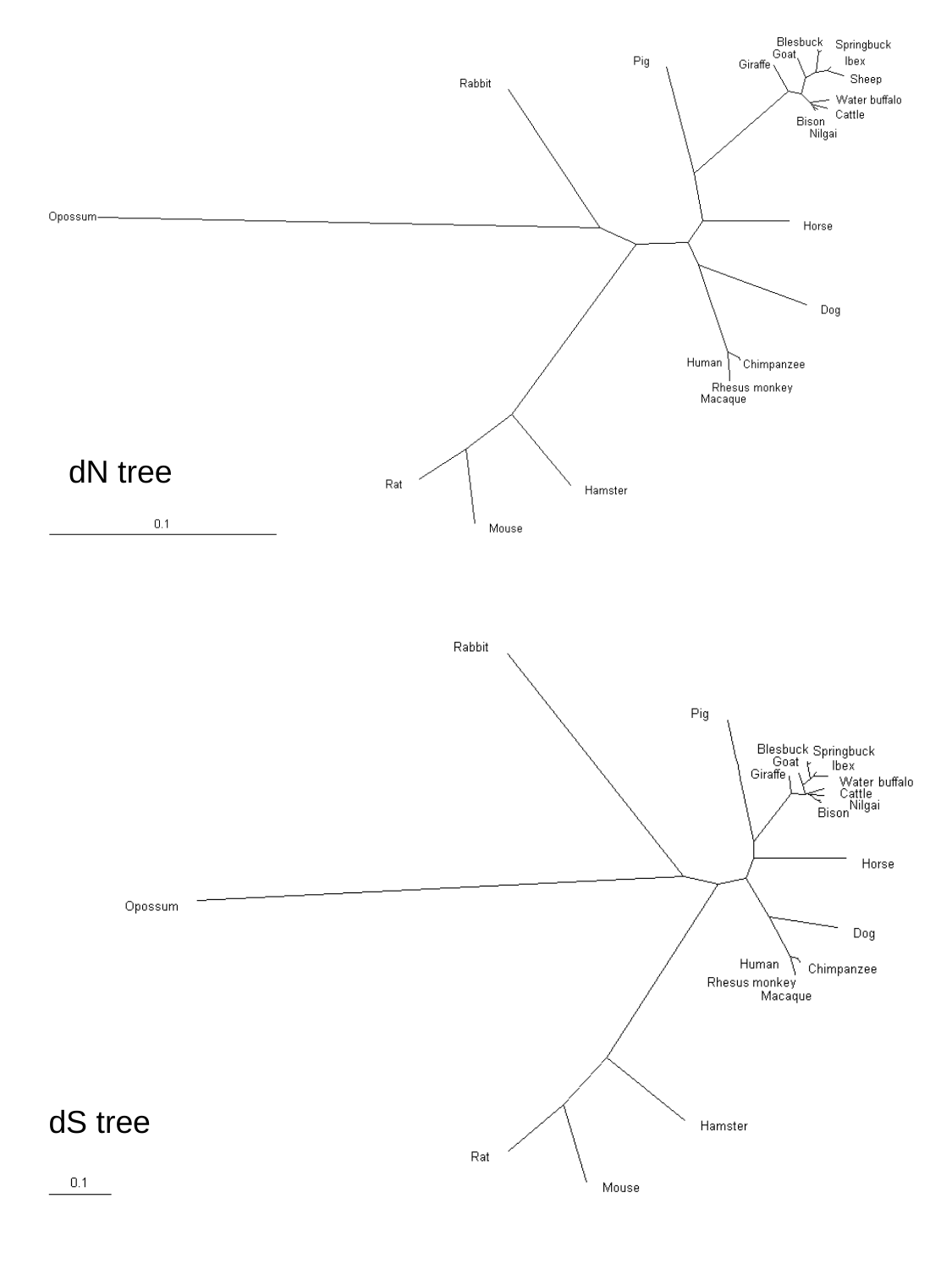

dN tree
dS tree

Supplement: Additional file 4 — Unrooted radial phylogenetic trees of the analysed TLR2 sequences. The dN tree displays the relationship between the sequences using non-synonymous exchanges only, the dS tree is limited on synonymous exchanges. The given scale represents a distance of 0.1 nucleotide exchanges per codon. [file 1471-2148-8-288-S4.ppt]
